# Effect of Extrusion Temperature on the Physico-Mechanical Properties of Unidirectional Wood Fiber-Reinforced Polylactic Acid Composite (WFRPC) Components Using Fused Deposition Modeling

**DOI:** 10.3390/polym10090976

**Published:** 2018-09-02

**Authors:** Teng-Chun Yang

**Affiliations:** Department of Forestry, National Chung Hsing University, Taichung 402, Taiwan; tcyang.04@nchu.edu.tw; Tel.: +886-4-22840345 (ext. 150)

**Keywords:** wood fiber, polylactic acid (PLA), 3D printing, fused deposition modeling (FDM), extrusion temperature, physico-mechanical properties

## Abstract

Wood fiber-reinforced polylactic acid (PLA) composites (WFRPCs) were used as a filament to manufacture the unidirectional WFRPC components by means of fused deposition modeling (FDM). The physico-mechanical properties of the WFRPC components printed at different extrusion temperatures (200, 210, 220, and 230 °C) were determined. The results revealed that most of the physical properties (moisture content, surface roughness, water absorption rate, and thickness swelling rate) of the printed WFRPC component were not significantly influenced by extrusion temperature, while its density and color difference increased as the extrusion temperature increased. Additionally, the tensile and flexural properties of the FDM-printed WFRPC component decreased when the extrusion temperature was more than 200 °C, whereas the compressive strength and internal bond strength increased by 15.1% and 24.3%, respectively, when the extrusion temperature was increased from 200 to 230 °C. Furthermore, scanning electronic microscopy (SEM) demonstrated that the fracture surface of the tensile component printed at a higher extrusion temperature exhibited a better compatibility at fiber/PLA interfaces and good adhesion between the extruded filament segments. These results indicate that the FDM printing process using different extrusion temperatures has a substantial impact on the surface color, density, and mechanical properties of the printed WFRPC component.

## 1. Introduction

In the last decade, additive manufacturing (AM), defined by ASTM F2792, has been a promising technology in various applications, such as aeronautics, civil engineering, automotive engineering, and medicine. Compared to traditional subtractive manufacturing, AM enables the automatic fabrication of products or functional components with complex shapes at a low manufacturing cost. Various commercially available AM methods include fused deposition modeling (FDM), inkjet printing (IP), selective laser sintering (SLS), laminated object manufacturing (LOM), and stereolithography (STL) [1]. Among these methods, FDM has recently gained popularity and achieved widespread use as the manufacturing process of desktop 3D printers. Many studies have reported that FDM allows solid parts with a 3D geometry to be formed by assembling successive layers of conventional or biodegradable thermoplastic material, such as polypropylene (PP), acrylonitrile butadiene styrene (ABS), and polylactic acid (PLA) [2,3,4,5,6,7,8,9,10]. Among raw materials of filaments, biobased PLA, which is made from renewable agricultural materials, has shown great potential to replace petroleum-based plastics due to its high mechanical properties and biodegradability. However, wider applications of PLA are limited by drawbacks, such as brittleness and a low thermal resistance. To overcome these drawbacks, natural fibers can be used as reinforcement to improve the characteristics of PLA since they have numerous positive effects: low density, high specific properties, renewability, biodegradability, and low cost [11,12,13]. Therefore, PLA composites reinforced with natural fibers have become increasingly attractive and have been studied due to their specific mechanical properties, suitable end-of-life management, and controlled environmental footprint [14]. Le Duigou et al. [15] presented that the water absorption rate, length swelling rate, and tensile properties of FDM-printed PLA composites that consist of wood fibers (10–20 wt %) were dependent on printing width and orientation.

A few studies have reported that the quality and mechanical properties of FDM-printed PLA components are significantly influenced by various processing parameters, such as layer thickness, feed rate, infill density and pattern, printing speed, extrusion temperature, and printing orientation [4,5]. The effect of layer thickness on the mechanical properties of PLA components was measured by Chacón et al. and Tymrak et al. [4,5]. They reported that the increase in tensile strength and the decrease in flexural strength of FDM-printed PLA products were observed as the layer thickness increased. Chacón et al. [4] showed that the tensile and flexural strengths of upright-printed PLA components decreased with the increase in feed rate, and they also indicated that this parameter impacted printing time and manufacturing cost. Furthermore, Ullu et al. [6] highlighted the effect of printing orientation on aspects of printed components, including surface roughness, dimensional accuracy, printing time and cost, and mechanical properties. The results indicated that mechanical properties of FDM-printed PLAs are highly dependent on printing orientation due to the significant changes in various structures of printed components. Several studies have shown that the strongest tensile performances of printed components are obtained when the printing orientation of the filament segment is parallel to the direction of the applied force [5,7,8]. Additionally, the influences of the infill pattern and density and the printing speed on the tensile properties and impact behavior of FDM-printed PLA components were explored by Tsouknidas et al. [9]. On the other hand, research concerning the mechanical properties of printed components with various extrusion temperatures has been examined [10], but not extensively investigated. Temperature, one of the primary effective processing parameters of FDM, influences the rheological behavior, crystallinity, deformation, and interlayer bonding strength of polymeric filament segments and further indirectly influences FDM printability and macromechanical properties of the printed components [10,16,17,18]. Furthermore, temperature also significantly affects the color and physico-mechanical properties of wood fibers due to changes in the chemical compositions of lignocellulosics, such as the increases in lignin-carbohydrate cross-linking and carbohydrate depolymerization and the decomposition of hemicellulose and lignin [19,20]. Among the chemical compositions, hemicellulose and lignin are relatively unstable compared to cellulose when the heating temperature is above 180 °C [19,21,22]. During the FDM printing process, temperature is generally set in the range of 200–230 °C; therefore, properties of the components that are printed from a wood fiber-reinforced PLA composite (WFRPC) are clearly changed. However, to the best of my knowledge, there is little available information on the effect of extrusion temperature on characteristics of FDM-printed WFRPCs. Hence, the purpose of the present study was to investigate the physical and mechanical performances of the WFRPC components printed at different extrusion temperatures.

## 2. Experimental

### 2.1. Materials

Commercial WFRPC filament (EasyWood^TM^) with a diameter of 1.75 mm was purchased from Formfutura BV (Gelderland, the Netherlands). The filament is a PLA matrix reinforced with approximately 40 wt % cedar fibers. Its density, melting point (MP), and melt flow index (MFI) were 1.20 g/cm^3^, 145 ± 10 °C, and 4.5 g/10 min, respectively.

### 2.2. 3D Printing of Unidirectional WFRPC Components

**Figure 1:**
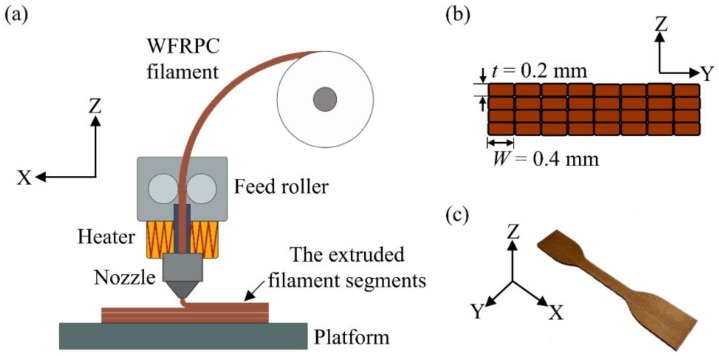
(**a**) Schematic of a customized FDM system; (**b**) the cross-section of the WFRPC component printed with the filament segments; (**c**) appearance of the FDM-printed tensile sample (extrusion temperature: 200 °C).

Unidirectional WFRPC components were fabricated using an FDM printer (Creator Pro, Flashforge Corp., Jinhua, China) with a 0.4 mm nozzle size. Given the basic setting in the customized FDM system, the line-by-line and layer-by-layer movements of the nozzle during the assembly of the extruded filament segments are depicted in Figure 1a. In this study, Flashprint software (Version 3.17.0, Flashforge Corp., Jinhua, China) was used to control the processing parameters and command the printer. All samples were printed with a rectilinear filling pattern to orient parallelly along the printing axis (X-axis) and without a contour. As shown in Figure 1b, the structural component of a WFRPC sample was fabricated by the filament segments with a printing width (*W*) of 0.4 mm and a layer thickness (*t*) of 0.2 mm. Four different extrusion temperatures (200, 210, 220, and 230 °C) were used to print the samples, which were designated as WFRPC_200_, WFRPC_210_, WFRPC_220_, and WFRPC_230_, respectively. For example, the appearance of the tensile sample printed with the extrusion temperature of 200 °C is shown in Figure 1c. For all conditions, the temperature of the heating platform and the printing speed was constant at 50 °C and 30 mm/s, respectively.

### 2.3. Determining the Printed Component Properties

The density, moisture content (MC), water absorption rate (WAR), and thickness swelling rate (TSR) were determined according to the ASTM D1037 (sample size: 36 mm × 12.5 mm × 6 mm). The ASTM D638, ASTM D790, and ASTM D695 methods were applied for determining the tensile properties, flexural properties, and compressive properties of the printed samples, respectively. The tensile properties, including the tensile strength (TS) and tensile modulus (TM), were assessed with type IV dumbbell-shaped samples at a loading speed of 5 mm/min and a span of 65 mm. The modulus of rupture (MOR) and modulus of elasticity (MOE) were obtained using a three-point bending test with a loading speed of 1.28 mm/min and a span of 48 mm (sample size: 80 mm × 12 mm × 3 mm). The compressive strength (CS) was determined on samples with dimensions of 1.2 mm × 1.2 mm × 2.4 mm at a compressive speed of 1.3 mm/min. The internal bond strength (IBS) was evaluated by a through-thickness tensile test at a tensile speed of 2 mm/min (sample size: 20 mm × 20 mm × 12 mm). Prior to testing, all samples were conditioned at 20 °C and 65% relative humidity for two weeks.

### 2.4. Thermogravimetric Analysis (TGA)

Residual weight (RW) and differential RW were recorded using a PerkinElmer Pyris 1 TG analyzer (PerkinElmer, Shelton, CT, USA). Approximately 3 mg of WFRPC filament sample was weighed in a platinum pan and operated under a continuous flux of nitrogen at a flow rate of 20 mL/min. The sample was heated from 50 to 600 °C with a 10 °C/min ramp.

### 2.5. Scanning Electron Microscopy (SEM)

The fracture surfaces of all tensile samples were examined by a JEOL JSM6330F SEM (JEOL Ltd., Tokyo, Japan) equipped with a field emission gun and an acceleration voltage of 3.0 kV. All samples were dried and sputtered with platinum before testing.

### 2.6. Surface Color of the Printed Components

A spectrophotometer (Minolta CM-3600d, Minolta Co., Tokyo, Japan) under a D65 light source was used to measure the CIE L*a*b* colors on the surface of the printed WFRPC component. Therein, L* is the value of the white/black axis, a* is the value of the red/green axis, b* is the value on the yellow/blue axis, and ΔE* is the color difference (ΔE* = [(ΔL*)^2^ + (Δa*)^2^ + (Δb*)^2^]^1/2^).

### 2.7. Surface Roughness of the Printed Components

The surface topographies of the printed WFRPC components were examined using an optical profilometer (Sensofar S Neox, Sensofar, Terrassa, Spain) in confocal mode with a 20× objective. The Z-scan thickness and the pixel size were 60 μm and 1.4 μm/pixel, respectively. The area of the surface topography for each sample was 4.4 mm × 4.3 mm. The average surface roughness (*R*_a_) was evaluated according to ISO 4287.

### 2.8. Statistical Analysis

All the results are expressed as the mean ± the standard deviation (SD). The significance of the differences was calculated using Scheffe’s test; *p* < 0.05 was considered to be significant.

## 3. Results and Discussion

### 3.1. Thermal Properties of a WFRPC Filament

**Figure 2:**
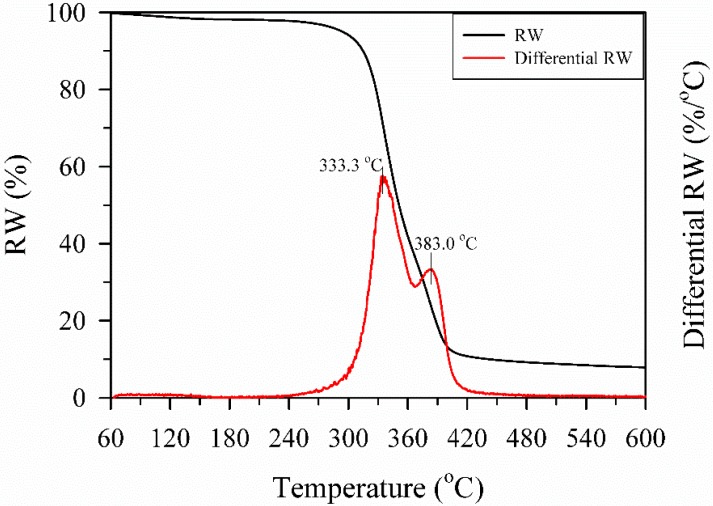
The RW and differential RW curves of the WFRPC filament obtained using TGA.

Prior to FDM printing, the thermal characterization of a filament is essential since its thermal behavior is capable of providing a printing window to understand the properties of a printed component. Figure 2 shows the RW and differential RW curves of a WFRPC filament against temperature. The RW curve showed that the weight started to decrease at 210 °C, and weight loss was obvious above 240 °C. Additionally, the differential RW curves were separated into three stages, with the first range of 60–120 °C, the second range of 210–370 °C, and the third range of 370–480 °C. It is well known that the first stage corresponds to water evaporation in the wood fibers. Órfão et al. [23] reported that during the second stage, the total decomposition of hemicellulose and cellulose and the partial decomposition of lignin simultaneously occur; during the third stage, the decomposition of the remaining lignin and the combustion of the residues take place. Two remarkable peaks were observed at approximately 333.3 and 383.0 °C. The former peak is associated with the main decomposition of cellulose, and the latter peak with the decomposition of the PLA matrix [24]. These results illustrated that the thermal degradation of wood fibers could occur during the FDM printing process with an extrusion temperature in the range of 200–230 °C, which is generally suggested to produce a WFRPC component.

### 3.2. Physical Properties of the Printed Components

**Figure 3:**
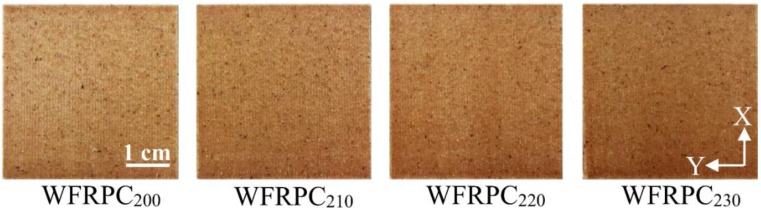
Surface appearances of the FDM-printed WFRPC components extruded at different temperatures.

Color measurement can indicate the degree of thermal degradation of the WFRPC filament during the FDM printing process. As shown in Figure 3, the color traits on the surfaces of the printed samples were influenced by an increase in extrusion temperature. The surface color was light brown for WFRPC_200_, and then it changed to dark brown for WFRPC_230_. The result indicated that the colors of the printed WFRPC components became darker as the extrusion temperature increased. The data in Table 1 indicate the effect of extrusion temperature on the color parameters of the FDM-printed WFRPC components. The L* value decreased from 57.3 (WFRPC_200_) to 52.9 (WFRPC_230_), while the a* value increased from 13.7 (WFRPC_200_) to 14.4 (WFRPC_230_). Simultaneously, the b* values of all samples were almost constant in the range of 22.4 to 22.7. The results showed that a decrease in the L* value and an increase in the a* value were observed as the extrusion temperature increased. In contrast, the b* value was not influenced by temperature. Additionally, compared to that of WFRPC_200_, the color difference (ΔE*) of WFRPC_230_ increased from 1.1 to 3.9 by increasing the extrusion temperature from 210 to 230 °C. This result indicated that the extrusion temperature affected the color of wood fibers in the WFRPC component. Previous studies reported that the color variation of the thermally treated wood is primarily caused by changes in the polysaccharides [25,26]. Kačíková et al. [27] and Bourgois et al. [28] stated that a decrease in L* and an increase in ΔE* are attributed to hemicellulose degradation. Bekhta and Niemz [25] reported that the darker color of wood after thermal treatment resulted from the changes in extractives, the formation of products from hemicellulose decomposition, and the formation of oxidation products, etc.

**Figure 4:**
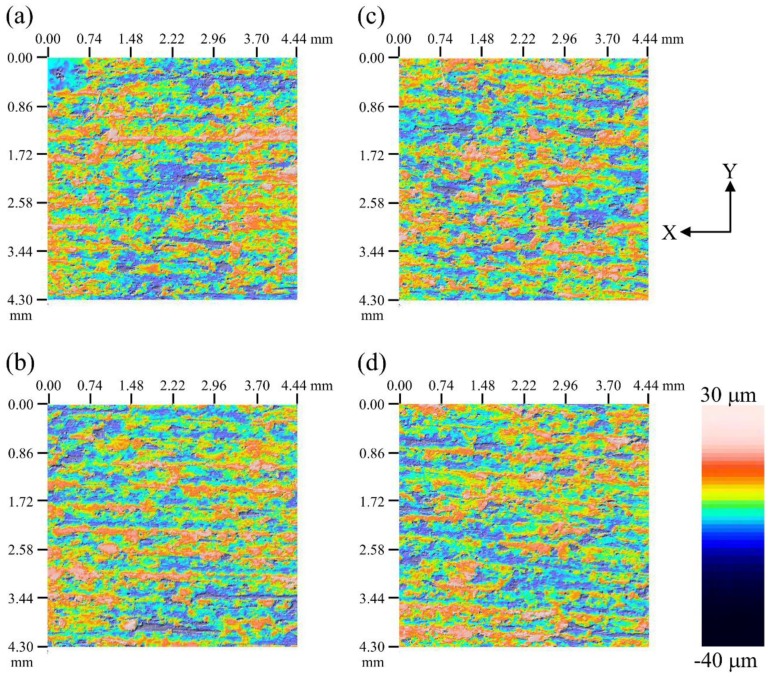
The surface topographies of the FDM-printed WFRPC components extruded at different temperatures obtained by the optical profilometer. (**a**) WFRPC_200_; (**b**) WFRPC_210_; (**c**) WFRPC_220_; and (**d**) WFRPC_230_.

Several physical properties of the FDM-printed WFRPC components with different extrusion temperatures are listed in Table 2. The weight (*w*) value of the printed sample significantly increased from 2.79 g (WFRPC_200_) to 2.91 g (WFRPC_230_), while its volume (*V*) was not significantly different among all samples in the range of 2.70–2.73 cm^3^. According to a previous study by Hwang et al. [29], the viscosity of the polymeric filament reduced as the temperature increased. Hence, the *w* value of the printed sample was increased by reducing the viscosity of the WFPRC filament as it was extruded at a higher temperature. As expected, WFRPC_230_ exhibited the highest density (1065 kg/m^3^) among all samples since it had the highest *w* value per volume. Additionally, the MC values of the printed samples were approximately 1.8%–2.1%, and no significant difference was noted among all samples. As shown in Figure 4, the surface topographies of the FDM-printed WFRPC components with different extrusion temperatures were evaluated using the optical profilometer. The average roughness (Ra) values on the surfaces of all samples were determined in the range of 6.0–6.3 μm (Table 2). The result demonstrated that the Ra values were not influenced by the extrusion temperature. Furthermore, after 24 h of soaking in water, the water absorption rate (WAR) and thickness swelling rate (TSR) of all samples were in the ranges of 2.6% to 3.1% and 0.8% to 1.1%, respectively. The water absorption of the WFRPC is mainly due to the hydrophilic nature of the wood fibers [15,30]. In addition, the gaps at the interfaces of filament segments and the porosities that are formed by FDM printing promote water absorption and diffusion into the samples [15]. As stated by previous studies [31,32], the polymer composites with the thermally treated wood fibers showed lower WAR and TSR compared to those of composites with nontreated fibers due to the modification in the chemical composition and structure of wood fibers after heat treatment. However, the values after the 24 h soaking test exhibited no significant difference among all components printed with different extrusion temperatures in this study. This phenomenon may be accounted for by the similar wettability of the PLA matrix on the surfaces of wood fibers among all samples.

### 3.3. Mechanical Properties of the Printed Components

**Figure 5:**
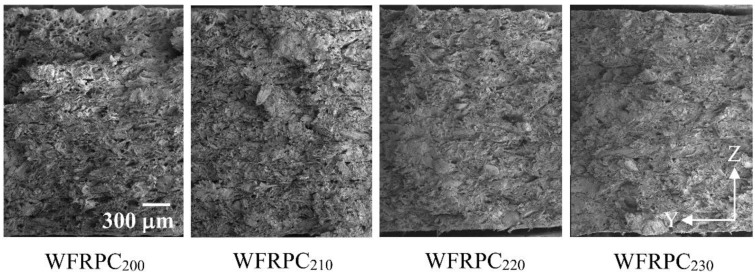
SEM micrographs for failure cross-sectional surfaces of the FDM-printed WFRPC components extruded at different temperatures.

The effect of the extrusion temperature on mechanical responses of the FDM-printed WFRPC components is shown in Table 3. The WFRPC_200_ showed tensile strength (TS) and tensile modulus (TM) values of 20.0 and 1802 MPa, respectively. The TS value decreased by approximately 9.5% (18.1 MPa) with an increasing extrusion temperature up to 220 °C and then leveled off. Additionally, the TM value significantly decreased in the range of 1711–1717 MPa when the extrusion temperature was more than 200 °C. For flexural properties, the MOR and MOE of WFRPC_200_ were 35.2 and 1928 MPa, respectively. Once the extrusion temperature exceeded 200 °C, the MOR reduced to 33.2–33.7 MPa, whereas the MOE noticeably decreased to 1557 MPa with an increasing temperature up to 230 °C. Compared to those of the WFRPC_200_, the higher extrusion temperature led to reductions of 4.3%–8.5% and 6.3%–19.2% in MOR and MOE, respectively, depending on the selected temperature. These results demonstrated that the WFRPC_200_ exhibited the highest tensile and flexural properties among all samples. However, these properties of the FDM-printed WFRPC components decreased when the extrusion temperature was more than 200 °C. The loss in the tensile and flexural properties can be attributed to the formation of acidic products from hemicellulose degradation when the WFRPC filament was fed through the heater. These acids cause the depolymerization and shortening of the cellulose polymer and the cleavage of C–C and C–O linkages at the intrapolymer level in wood fibers [33,34]. Several studies have stated that heat treatment using high temperature leads to decreased flexural properties of wood, wood-based products, and WFRPCs [32,35,36,37,38]. Figure 5 presents SEM micrographs of the cross-sectional surfaces of the FDM-printed samples with various extrusion temperatures after tensile testing. In the case of the WFRPC_200_, pull-outs of wood fibers occurred more often among all samples. This result indicated that there is poor adhesion between wood fibers and the PLA matrix. Previous studies have reported that incompatibility between the hydrophilic lignocellulosics and hydrophobic thermoplastics was found in wood‒PLA composites [39,40]. With increasing extrusion temperature up to 230 °C (WFRPC_230_), the fiber breakages and some pull-outs can be clearly observed at the fracture surface. This result suggests that wood fibers were further heat treated when the filament was fed through the heater at a higher temperature. Therefore, this finding could be related to two conditions. One condition is the decline in mechanical properties of wood fibers due to changes in their chemical composition, as described above [33,34,41]. The other condition is the improvement in compatibility of the wood fibers to increase the fiber/PLA bonding interactions, which is a requirement for the wide differences in the polarity of their surfaces [42,43]. The increase in bonding results in a smooth transmission of stress due to better adhesion of the two surfaces.

**Figure 6:**
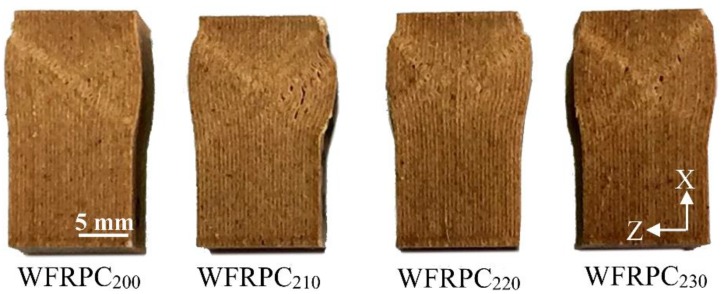
Appearances of compressive failure of the FDM-printed WFRPCs extruded at different temperatures.

Moreover, the WFRPC_200_ exhibited the lowest CS value (28.5 MPa) among all samples. As the extrusion temperature increased to 230 °C, the CS value significantly increased by 15.1% (32.8 MPa) compared to that of the WFRPC_200_. As shown in Figure 6, the damage evolution of all samples was caused by the buckling and kinking of filament segments, leading to deboning between their interfaces. When the FDM-printed WFRPC component was subjected to the longitudinal compression along the printing orientation, its load-carrying capacity was mainly influenced by the internal bond strength between filament segments, which is related to transverse deformation. As shown in Table 3, the WFRPC_230_ exhibited the highest IBS value (4.6 MPa) among all samples. This phenomenon is mainly attributable to the strong interlayer bonding between filament segments and the better compatibility of fiber/PLA interfaces compared to those of the WFRPC_200_ (Figure 5), leading to the higher CS and IBS values. As mentioned above, these results indicate that the FDM printing with a high extrusion temperature (> 200 °C) decreases the tensile and flexural properties and increases the compressive strength and internal bond strength of the component.

## 4. Conclusions

WFRPC components were prepared by FDM printing at different extrusion temperatures in the range of 200–230 °C. Compared to that of the WFRPC_200_ samples, an increase in ΔE* of the printed samples was observed as the extrusion temperature increased. The WFRPC_230_ sample showed the highest *w* value, resulting in the highest density among all samples since the viscosity of the WFRPC filament was reduced at a higher extrusion temperature. However, there was no significant difference in the MC and Ra values among all printed samples. On the other hand, the WAR and TSR of the components printed at a higher extrusion temperature (> 200 °C) also exhibited no significant difference compared to those of WFRPC_200_. In addition, the extrusion temperature clearly affected the mechanical properties of the printed WFRPC components. The tensile (TS and TM) and flexural (MOR and MOE) properties of the component obtained using the FDM decreased with an increasing extrusion temperature due to further thermal degradation of wood fibers. Moreover, the CS and IBS values of the printed samples increased as the extrusion temperature increased from 200 to 230 °C. In the SEM micrographs, the fracture surfaces of the tensile samples printed at a higher extrusion temperature showed better compatibility at fiber/PLA interfaces and stronger interlayer bonding between filament segments. Accordingly, the performances of the WFRPC component were influenced by the extrusion temperature. These results provide information on the FDM-printed manufacturing of controllable and optimized WFRPC components for specific applications, such as thermal- and mechanical-resistant jointing or supporting materials.

## Figures and Tables

**Table 1:** The surface color parameters of the FDM-printed WFRPC components extruded at different temperatures.

Code	L*	a*	b*	ΔE*
WFRPC_200_	57.3 ± 0.6 ^a^	13.7 ± 0.1 ^c^	22.5 ± 0.1 ^a^	-
WFRPC_210_	56.1 ± 1.0 ^a,b^	14.0 ± 0.2 ^b,c^	22.7 ± 0.2 ^a^	1.1 ± 0.4 ^b^
WFRPC_220_	54.0 ± 0.8 ^b,c^	14.2 ± 0.2 ^a,b^	22.4 ± 0.3 ^a^	2.7 ± 0.9 ^a^
WFRPC_230_	52.9 ± 0.2 ^c^	14.4 ± 0.1 ^a^	22.4 ± 0.1 ^a^	3.9 ± 0.1 ^a^

Values are the mean ± SD (*n* = 3). Different letters (a, b, and c) within a column indicate a significant difference at *p* < 0.05.

**Table 2:** The physical properties of the FDM-printed WFRPC components extruded at different temperatures.

Code	*w* (g)	*V* (cm^3^)	Density (kg/m^3^)	MC (%)	*R*_a_ (μm)	24 h of Soaking	
						WAR (%)	TSR (%)
WFRPC_200_	2.79 ± 0.08 ^b^	2.71 ± 0.04 ^a^	1032 ± 17 ^b^	2.1 ± 0.3 ^a^	6.0 ± 0.6 ^a^	3.1 ± 0.5 ^a^	0.8 ± 0.1 ^a^
WFRPC_210_	2.83 ± 0.04 ^a,b^	2.70 ± 0.01 ^a^	1048 ± 12 ^a,b^	1.9 ± 0.2 ^a^	6.2 ± 0.6 ^a^	2.9 ± 0.2 ^a^	1.1 ± 0.1 ^a^
WFRPC_220_	2.81 ± 0.07 ^b^	2.70 ± 0.02 ^a^	1041 ± 17 ^b^	1.8 ± 0.2 ^a^	6.0 ± 0.7 ^a^	3.1 ± 0.2 ^a^	0.9 ± 0.1 ^a^
WFRPC_230_	2.91 ± 0.01 ^a^	2.73 ± 0.01 ^a^	1065 ± 6 ^a^	1.9 ± 0.2 ^a^	6.3 ± 0.5 ^a^	2.6 ± 0.2 ^a^	1.0 ± 0.3 ^a^

Values are the mean ± SD (*n* = 6). Different letters (a and b) within a column indicate a significant difference at *p* < 0.05.

**Table 3:** The mechanical properties of FDM-printed WFRPC components extruded at different temperatures.

Code	Tensile properties		Flexural properties		CS (MPa)	IBS (MPa)
	TS (MPa)	TM (MPa)	MOR (MPa)	MOE (MPa)		
WFRPC_200_	20.0 ± 0.5 ^a^	1802 ± 32 ^a^	35.2 ± 1.0 ^a^	1928 ± 66 ^a^	28.5 ± 0.4 ^c^	3.7 ± 0.3 ^b^
WFRPC_210_	19.5 ± 1.0 ^a^	1717 ± 63 ^b^	33.7 ± 1.6 ^a,b^	1699 ± 84 ^b,c^	31.2 ± 0.6 ^b^	3.6 ± 0.4 ^b^
WFRPC_220_	18.1 ± 0.4 ^b^	1711 ± 39 ^b^	32.2 ± 1.4 ^b^	1806 ± 75 ^a,b^	30.4 ± 0.5 ^b^	4.0 ± 0.3 ^a,b^
WFRPC_230_	18.0 ± 0.1 ^b^	1713 ± 15 ^b^	32.8 ± 1.4 ^a,b^	1557 ± 128 ^c^	32.8 ± 0.5 ^a^	4.6 ± 0.4 ^a^

Values are the mean ± SD (*n* = 6). Different letters (a, b, and c) within a column indicate a significant difference at *p* < 0.05.
